# *In vitro* Activity of Repurposed Nitroxoline Against Clinically Isolated Mycobacteria Including Multidrug-Resistant *Mycobacterium tuberculosis*

**DOI:** 10.3389/fphar.2022.906097

**Published:** 2022-05-26

**Authors:** Ada Marie Hoffmann, Martina Wolke, Jan Rybniker, Georg Plum, Frieder Fuchs

**Affiliations:** ^1^ Institute for Medical Microbiology, Immunology and Hygiene, University of Cologne, Medical Faculty and University Hospital of Cologne, Cologne, Germany; ^2^ Department I of Internal Medicine, Faculty of Medicine and University of Cologne, Cologne, Germany; ^3^ German Center for Infection Research (DZIF), Partner Site Bonn Cologne, Cologne, Germany; ^4^ Department of Microbiology and Hospital Hygiene, Bundeswehr Central Hospital Koblenz, Koblenz, Germany

**Keywords:** MDR, XDR, quinoline, 8-hydroxy-quinoline, genitourinary TB, UTI, BCGitis

## Abstract

Antimicrobial treatment options for mycobacterial infections are limited due to intrinsic resistance and the emergence of acquired resistance in *Mycobacterium tuberculosis*. Isolates resisting first- and second line drugs are raising concerns about untreatable infections and make the development of new therapeutic strategies more pressing. Nitroxoline is an old oral antimicrobial that is currently repurposed for the treatment of urinary tract infection (UTI). In this study, we report the *in vitro* activity of nitroxoline against 18 clinical isolates of *M. tuberculosis* complex (MTBC) (*M. tuberculosis* N = 16, *M. bovis* BCG N = 1, *M. bovis* sp. *bovis* N = 1). Since nitroxoline achieves high concentrations in the urinary tract, we included all MTBC-isolates from urinary samples sent to our laboratory between 2008 and 2021 (University Hospital of Cologne, Germany). Isolates from other sources (N = 7/18) were added for higher sample size and for inclusion of drug-resistant *M. tuberculosis* isolates (N = 4/18). Based on our clinical routine the fluorescence-based liquid media system BACTEC MGIT 960 was used for susceptibility testing of nitroxoline and mainstay antitubercular drugs. Nitroxoline yielded a MIC_90_ of 4 mg/L for MTBC. In all *M. tuberculosis* isolates nitroxoline MICs were at least two twofold dilutions below the current EUCAST susceptibility breakpoint of ≤16 mg/L (limited to *E. coli* and uncomplicated UTI). *In vitro* activity of nitroxoline can be considered excellent, even in multidrug-resistant isolates. Future studies with *in vivo* models should evaluate a potential role of nitroxoline in the treatment of tuberculosis in the era of drug resistance.

## Introduction

The tuberculosis epidemic is ongoing and complicated by the increasing number of infections with drug resistant mycobacteria. Multidrug resistance (defined as resistance to isoniazid and rifampicin) makes the treatment even more difficult and is associated with longer treatment duration, higher costs, more toxic drugs and a lower cure rate (WHO, 2020). For novel therapeutic strategies in the fight against *Mycobacterium tuberculosis* (MTB), the repurposing of antimicrobials approved for the therapy of other infections is warranted (Mourenza et al., 2020).

Nitroxoline (8-hydroxy-5-nitroquinoline) is a quinoline derivate that is structurally unrelated to other antimicrobials. First described in the 1950s (Petrow and Sturgeon, 1954) it is currently recommended as one first line drug for the oral treatment of uncomplicated urinary tract infections (uUTI) in the German uUTI treatment guideline (250 mg capsules three times daily) (Kranz et al., 2017). The antimicrobial activity derives on chelation of bivalent cations, resulting in a reduced bacterial adhesion to epithelial cells (Pelletier et al., 1995; Naber et al., 2014; Oliviero et al., 1990) and diverse effects on cellular enzymatic pathways including the inhibition of RNA-polymerases (Fraser and Creanor 1974). Activity against different multidrug-resistant pathogens and potent inhibition of biofilms has recently led to regained interest in nitroxoline repurposing (Abouelhassan et al., 2017; Fuchs and Hamprecht 2019; Fuchs et al., 2019; Fuchs et al., 2021; Joaquim et al., 2021; Fuchs et al., 2022a; Fuchs et al., 2022b). Despite over 50 years of clinical use, resistance to nitroxoline has rarely been reported (Kresken and Körber-Irrgang, 2014). The current clinical breakpoint (susceptible, ≤ 16 mg/L) was introduced by EUCAST in 2016 and is limited to *E. coli* and uUTI (EUCAST, 2021). High nitroxoline concentrations in urine and prostate tissue can be achieved with oral therapy compared to lower systemic concentrations (Dufour and Bollack, 1979; Mrhar et al., 1979; Wijma et al., 2018). Activity against some mycobacterial quality control strains (*Mycobacterium bovis* BCG ATCC 35734 and *M. tuberculosis* H37rv) has been demonstrated (Odingo et al., 2019; Shah et al., 2016; Murugasu-Oei and Dick, 2001) yet data on clinical isolates, especially with respect to otherwise drug resistant MTB are lacking. In this study, we therefore assessed nitroxoline MICs of clinically isolated mycobacteria including MDR-TB isolates to investigate repurposing of nitroxoline for mycobacterial infections in the era of antimicrobial resistance.

## Material and Methods

### Isolates

The collection consists of 18 clinical isolates that were previously characterized as *M. tuberculosis* complex (MTBC) (16/18 MTB, 1/18 *M. bovis* sp. *bovis,* 1/18 *M. bovis* BCG). Most isolates (11/18) were isolated from urinary specimens, including one isolate from a patient suffering from BCGitis after intra-bladder Instillation of Calmette-Guérin *Bacillus*. Seven (7/18) MTB-isolates were included from other clinical sources [biopsies (N = 3), (soft tissue, lymph node, bone marrow), respiratory materials (N = 3), testicular swab (N = 1)]. All isolates were from specimen sent to our clinical microbiology laboratory (University hospital of Cologne, Germany) between 2008 and 2021 and identification was previously performed as part of clinical routine according to microbiological quality standards (CLSI, 2018a; CLSI, 2018b).

### Susceptibility Testing

Susceptibility testing of isoniazid, rifampicin, ethambutol, streptomycin and pyrazinamide was conducted in clinical routine using the fluorescence-based liquid media system BACTEC MGIT 960 (BD Diagnostics, Heidelberg, Germany) according to CLSI standards and the results were interpreted according to WHO breakpoints for all clinical MTBC isolates (Canetti et al., 1963; CLSI, 2018a; WHO, 2018). Four isolates were previously characterized as drug-resistant/multidrug-resistant (DR/MDR), therefore also second-line antitubercular drugs were tested, Table 1. A resistance to pyrazinamide tested in MGIT BD BACTEC was confirmed using molecular detection of *pncA* Gln10Pro-Mutation (Werngren et al., 2012; Tam et al., 2019).

**TABLE 1:** Susceptibility of drug- and multidrug-resistant *M. tuberculosis* isolates (1–4) (DR-/MDR-TB) (*n* = 4).

	1	2	3	4
	*M. tuberculosis* (MDR-TB)	*M. tuberculosis* (MDR-TB)	*M. tuberculosis* (MDR-TB)	*M. tuberculosis* (DR-TB)
Isoniazid	R	R	R	R
MIC in mg/L	>1.0	>1.0	>1.0	>1.0
Rifampicin	R	R	R	S
MIC in mg/L	>10.0	>10.0	>10.0	<1.0
Ethambutol	S	S	S	S
MIC in mg/L	5.0	<2.5	5.0	<2.5
Pyrazinamide^a^	S	R	S	S
Streptomycin	R	R	R	R
MIC in mg/L	>50.0	4.0	>50.0	>50.0
Amikacin	S	S	R	S
MHK in mg/L	<1.0	<1.0	16.0	<1.0
Capreomycin	S	S	S	S
MIC in mg/L	<1.25	2.5	<1.25	2.5
Kanamycin	S	S	R	S
MIC in mg/L	<2.5	<2.5	20.0	<2.5
Linezolid	S	S	S	S
MIC in mg/L	<0.5	<0.5	<0.5	<0.5
Levofloxacin	S	R	S	S
MIC in mg/L	<0.75	>3.0	<0.75	<0.75
Moxifloxacin	S	R	S	S
MIC in mg/L	<0.25	4.0	<0.25	<0.25
Para-Aminosalicylacid	S	S	R	S
MIC in mg/L	<2.0	<2.0	>8.0	<2.0
Rifabutin	R	R	R	S
MIC in mg/L	>2.0	2.0	>2.0	<0.5
Nitroxoline				
MIC in mg/L	4.0	4.0	4.0	4.0

a Pyrazinamide resistance was assessed by Gln10Pro-Mutation (Werngren et al., 2012; Tam et al., 2019).

For nitroxoline susceptibility testing pure nitroxoline powder (provided by Rosen Pharma, St. Ingbert, Germany) was dissolved in DMSO (Sigma Aldrich, Darmstadt, Germany). For further dilution in MGIT, standardized aliquots with a 2-fold dilution range (1-2-4-8-16-32 mg/L) were aseptically prepared. MIC values were determined using the MGIT BD BACTEC 960 connected to a BD EpiCenter Microbiology Data Management System (Becton Dickinson, Sparks, United States) equipped with a TBeXIST module for drug susceptibility testing (DST). DST was performed according to the manufacturers manual (Siddiqi and Rüsch-Gerdes, 2006; BD, 2010) based on clinical routine DST of standard antitubercular drugs. In brief, MGIT tubes were prepared with 0.8 ml MGIT BACTEC 960 SIRE Supplement, then 0.1 ml of drug solution and 0.5 ml of test strain suspension were added. A growth control was prepared in a drug-free tube with 0.1 ml of test strain suspension previously diluted into 10 ml sterile saline for a 1:100 dilution. The nitroxoline MIC was defined as the lowest drug concentration that kept growth under 100 growth units when the BACTEC 960 reader indicated 400 growth units for the drug-free control. Growth controls in each cycle were time controlled for each isolate and also compared to a drug free growth control containing DMSO dilutions to rule out confounding effects of the bactericidal solvent DMSO. The MTB wild-type H37Rv (ATCC 27294) was used as quality control strain.

## Results

Nitroxoline was active in all MTBC isolates (N = 18) with a MIC_90_ of 4 mg/L (range: 4-8 mg/L) irrespective of the species. The highest MIC of 8 mg/L was demonstrated for the *M. bovis* BCG isolate.

Nitroxoline MIC was 4 mg/L in all four drug-resistant or multidrug-resistant MTB-isolates. Table 1 summarizes all susceptibility results of the four drug resistant isolates (1-4) of which all were resistant to isoniazid and streptomycin. Isolate 1, 2 and 3 were additionally resistant to rifampicin and isolate 2 was also pyrazinamide resistant. Resistance to amikacin, kanamycin, para-aminosalicylacid and fluoroquinolones was also detected, Table 1.

Additional susceptibility results for all isolates are displayed in Table 2. In brief, one *M. bovis* sp. *bovis* was resistant to isoniazid and pyrazinamide but was susceptible to rifampicin, ethambutol and streptomycin. *M. bovis* BCG was susceptible to isoniazid, rifampicin, ethambutol and streptomycin. The H37Rv (ATCC 27294) strain, included for quality control, was in the expected range for standard drugs and yielded a nitroxoline MIC of 4 mg/L.

**TABLE 2:** Isolate overview stratified by species with corresponding results of drug susceptibility testing.

Isolate number	Species	Source of isolation	classification	Antimicrobial substances (S: susceptible, R: resistant according to WHO critical concentrations)					
				Isoniazid	Rifampicin	Ethambutol	Streptomycin	Pyrazinamid	Nitroxoline MIC mg/L
1	*M. tuberculosis*	lung tissue	MDR-TB	R	R	S	R	S	4
2	*M. tuberculosis*	biopsy flank	MDR-TB	R	R	S	R	R	4
3	*M. tuberculosis*	sputum	MDR-TB	R	R	S	R	S	4
4	*M. tuberculosis*	biopsy bone marrow	DR-TB	R	S	S	R	S	4
5	*M. tuberculosis*	urine	DS-TB	S	S	S	S	S	4
6	*M. tuberculosis*	urine	DS-TB	S	S	S	S	S	4
7	*M. tuberculosis*	urine	DS-TB	S	S	S	S	S	4
8	*M. tuberculosis*	urine	DS-TB	S	S	S	S	S	4
9	*M. tuberculosis*	urine	DS-TB	S	S	S	S	S	4
10	*M. tuberculosis*	testicle swap	DS-TB	S	S	S	S	S	4
11	*M. tuberculosis*	urine	DS-TB	S	S	S	S	S	4
12	*M. tuberculosis*	urine	DS-TB	S	S	S	S	S	4
13	*M. tuberculosis*	urine	DS-TB	S	S	S	S	S	4
14	*M. tuberculosis*	urine	DS-TB	S	S	S	S	S	4
15	*M. tuberculosis*	sputum	DS-TB	S	S	S	S	S	4
16	*M. tuberculosis*	urine	DS-TB	S	S	S	S	S	4
17	*M. bovis* BCG	urine	n.a.	S	S	S	S	-	8
18	*M. bovis* sp. bovis	lymph node	n.a.	R	S	S	S	-	4

MIC: minimal inhibitory concentration, DS-TB: drug susceptible TB; DR-TB: drug-resistant TB; MDR-TB: multidrug-resistant TB.

## Discussion

In mycobacterial infections isolates resisting conventional first- and second line antitubercular drugs are raising concerns, however only few novel drugs are being developed. Nitroxoline is an old oral antimicrobial with an approved efficacy and safety profile for treatment of UTI (Naber et al., 2014). The present study investigated the activity of nitroxoline against mycobacteria from clinical sources with a focus on urogenital isolates and with inclusion of drug-resistant MTB.

After oral administration, nitroxoline is rapidly absorbed from the gastrointestinal tract and undergoes conjugation in the liver (Mrhar et al., 1979). Most antimicrobial effects are attributed to the unconjugated form of nitroxoline, which was tested in this study (Wagenlehner et al., 2014). Since conjugated nitroxoline was not available for *in vitro* testing, specific antimicrobial effects of these molecules against mycobacteria remain unclear. However, concentrations of 5.4 mg/L for the unconjugated and 210.6 mg/L for the conjugated form have been demonstrated in urine after oral therapy with a dose of 250 mg q8h in a recent study (Forstner et al., 2018) exceeding the MICs assessed for all MTB isolates (including MDR-isolates).

In contrast to standard oral therapy, time responses in tissue and plasma were relatively low or quickly decreasing after parental administration of 3 g nitroxoline in a previous study (Mrhar et al., 1979). Overall, the current pharmacokinetic data of nitroxoline refers to old studies with rather small sample sizes, as reviewed recently (Wijma et al., 2018) and more research is needed. Of note, nitroxoline dose increase from 30 mg/kg twice a day to 240 mg/kg twice a day in a preclinical study with mice did not increase toxicity (measured by mice body weight and mice mortality) (Mirkovic et al., 2015). Therefore, future studies on nitroxoline pharmacokinetics should also consider if higher dosing may lead to higher tissue concentrations in body sites other than the urinary tract, which could have important implications for nitroxoline repurposing. Based on our findings, nitroxoline repurposing for the combination treatment of genitourinary tuberculosis should be considered and analyzed with *in vivo* models, especially with respect to rising antimicrobial resistance in extrapulmonary TB entities (Pang et al., 2019).

Our results are in line with the scarce data of previous studies about nitroxoline activity against mycobacteria, however due to differences in methodology comparability of MIC results is limited. One study investigated nitroxoline activity against the *M. bovis* BCG strain ATCC35734 demonstrating a MIC of 2 mg/L, which is two dilutions below our results for *M. bovis* BCG (Murugasu-Oei and Dick, 2001). In other studies, low nitroxoline MICs against the *M. tuberculosis* quality control strain H37rv were displayed (Odingo et al., 2019) including data on bactericidal effects in a macrophage infection model, highlighting a physiological relevance of these low MICs within the complex pathogenesis of tuberculosis (Shah et al., 2016). In our study, a larger set of clinical isolates was used to compare MICs of nitroxoline in drug susceptible and drug resistant isolates to evaluate nitroxoline particularly in difficult to treat MTBC isolates. Nitroxoline MICs were 4 mg/L in both, susceptible and multidrug-resistant isolates, therefore nitroxoline repurposing is particularly interesting in the current era of mycobacterial multidrug-resistance.

Our study has several limitations. Most importantly low MICs do not necessarily translate into clinical success *in vivo.* In general, despite prescription experiences for decades in some European countries (Naber et al., 2014) studies with clinical endpoints of nitroxoline therapy are rare and treatment failure in UTI has been reported (Forstner et al., 2018). It is therefore important to not overestimate *in vivo* capacities of nitroxoline based on *in vitro* findings, especially with respect to the complex intracellular pathogenesis of mycobacteria that includes deep tissue infection by entering and trafficking in host immune cells with formation of granulomatous focal lesions. How primarily urinary concentrated nitroxoline may affect this pathogenesis remains undetermined, also intracellular drug concentrations may vary from corresponding extracellular drug levels (e.g., serum). Since nitroxoline effects depend on ion chelation, the presence of ions *in vivo* may also affect nitroxoline activity (Shah et al., 2016). Nevertheless, the *in vitro* MICs are excellent even in MDR-MTB (MIC_90_ 4 mg/L) and relevant dose-dependent tissue concentrations were described for urine and prostate, highlighting a potential role of nitroxoline in combination therapy of genitourinary manifestations of tuberculosis, including BCGitis (Murugasu-Oei and Dick, 2001). Secondly, pharmacokinetics of nitroxoline is not limited to relevant urinary concentrations, since effective targeted drug delivery to specific tissues based on nanoparticles has already been demonstrated in cell culture assays and mice (Varshosaz et al., 2020; Hu et al., 2018). The potential of nitroxoline drug delivery to cells and tissues infected by mycobacteria outside the genitourinary tract should therefore be investigated in future studies especially since nanoparticle based approaches have already been investigated in mycobacterial infections (Johnson et al., 2005). Future studies should also analyze if currently known mechanisms of nitroxoline resistance [e.g., efflux pump mutations (Puértolas-Balint et al., 2020)] can be demonstrated in mycobacterial isolates with high nitroxoline MICs, if available.

To conclude, nitroxoline seems to be a promising candidate for drug repurposing against urogenital mycobacterial infections, especially considering the increasing incidence of multidrug-resistance in mycobacteria. Future studies should correlate these *in vitro* findings with clinical endpoints based on *in vivo* models.

## Data Availability

The original contributions presented in the study are included in the article/Supplementary Material, further inquiries can be directed to the corresponding author.
